# Expression of heat shock protein 70 in nasopharyngeal carcinomas: different expression patterns correlate with distinct clinical prognosis

**DOI:** 10.1186/1479-5876-10-96

**Published:** 2012-05-16

**Authors:** Man-Bo Cai, Xiao-Pai Wang, Jia-Xing Zhang, Hui-Qiong Han, Chao-Chun Liu, Jin-Xin Bei, Ruo-Jun Peng, Yi Liang, Qi-Sheng Feng, Hai-Yun Wang, Li-Zhen Chen, Sha Fu, Tiebang Kang, Jian-Yong Shao, Yi-Xin Zeng

**Affiliations:** 1 State Key Laboratory of Oncology in South China, Cancer Center, Sun Yat-Sen University, 651# Dongfeng Road East, Guangzhou 510060, China; 2 Cancer Research Institute, University of South China, Hengyang, China; 3 Department of Molecular Diagnostics, Cancer Center, Sun Yat-Sen University, 651# Dongfeng Road East, Guangzhou 510060, China; 4 Wadsworth Center, New York State Department of Health, Albany, NY, USA

**Keywords:** Nasopharyngeal carcinoma, Heat shock protein 70, Expression, Prognosis

## Abstract

**Background:**

Heat shock protein 70, a stress protein, has been implicated in tumor progression. However, its role in nasopharyngeal carcinoma (NPC) progression has not yet been clearly investigated.

**Methods:**

Immunohistochemistry (IHC) was employed to examine the expression patterns of Hsp70, human leukocyte antigen –A (HLA-A) in NPC tissue samples.

**Results:**

The expression of Hsp70 exhibited different spatial patterns among nuclear, membrane and cytoplasm in 507 NPC tumor tissues. Kaplan-Meier survival analysis demonstrated that different Hsp70 expression patterns are correlated with different patient outcomes. High membranal and cytoplasmic levels of Hsp70 predicted good survival of patients. In contrast, high nuclear abundance of Hsp70 correlated with poor survival. Moreover, the membranal and cytoplasmic levels of Hsp70 were positively correlated with levels of the MHC I molecule HLA-A.

**Conclusions:**

Different Hsp70 expression patterns had distinct predictive values. The different spatial abundance of Hsp70 may imply its important role in NPC development and provide insight for the development of novel therapeutic strategies involving immunotherapy for NPC.

## Background

Nasopharyngeal carcinoma (NPC) is a leading lethal malignancy with a high prevalence in Southeast Asia, especially in the Guangdong, Guangxi and Hong Kong areas in Southern China [1,2]. The incidence rate of NPC is 25–40 cases per 100,000 person-years in the Cantonese region of Southern China [3]. Most NPC tumor cells are poorly differentiated or undifferentiated and have high tendency to invade adjacent regions and metastasize to neck lymph nodes. Radiotherapy (RT) and concurrent chemoradiotherapy (CCRT) are standard treatments for NPC at early stage and advanced stage, respectively [4-6]. Therapeutic strategies have been studied aiming to improve the survival rate for advanced NPC. Recently, novel therapies based on molecular targets and neoadjuvant chemotherapy (NAC) followed by RT of NPC were promising for advanced lesions [7,8], though need to be validated in more trials. Conventional TNM staging has a strong prognostic implication for NPC [9]; and patients at early-stage are almost curable under RT, however, the prognosis remains poor in a significant number of patients with late-stage NPC [10] . Local failure and distant metastasis are major issues resulting in poor outcomes in NPC patients at advanced-stage [11]. Therefore, it is of clinical value to identify factors that allow for an early diagnosis and the prediction of prognosis and to discover novel therapeutic strategies.

During tumor development, cancer cells frequently encounter a variety of cytotoxic conditions, such as hypoxia and local hyperthermia [12,13]. Heat shock proteins (HSPs), also termed stress proteins, are induced by a variety of stresses, such as hyperthermia, and play pivotal roles in tumor cell survival after exposure to unfavorable insults [14]. Among the HSPs, Hsp70 mediates the protection of tumor cells from stress-induced lethal damage by interfering apoptotic pathways [15,16]. Furthermore, high cytosolic levels of Hsp70 are frequently associated with a negative clinical outcome and a higher frequency of metastasis [17]. Studies on NPC have shown that high serum levels of Hsp70 are strongly associated with T classification, metastasis and mortality, indicating a poor prognosis [18,19].

HSPs also have important roles in the immune system as carriers of tumor antigens [20,21]. Hsp70-chaperoned peptides derived from the cytosol of human tumors can activate a classical protective T-cell mediated immune response [22], which involves the uptake of HSP-peptide complexes by antigen-presenting cells (APCs) and the subsequent cross-presentation on MHC class I molecules to specific CD8^+^ T-cell populations [22] . In a study of NPC, the reconstituted complex with mycobacterial Hsp70 and EBV LMP2A-derived peptides has been shown with ability to elicit peptide-specific cytotoxic T-lymphocyte responses and anti-tumor immune cells [23]. Hsp70, therefore, appears to play dual roles in the progression of tumors. To date, however, the expression dynamics of Hsp70 tissues and the relevance to the clinicopathologic and prognostic significance have not been clearly investigated in NPC.

Here, we first examined Hsp70 expression patterns with immunohistochemical (IHC) staining of 507 paraffin-embedded NPC tumor tissues. Secondly, we analyzed the correlation between the Hsp70 expression level and the clinical factors and outcomes for NPC patients. Furthermore, we evaluated the possible role of Hsp70 in the progression of NPC tumors.

## Materials and methods

### Patients and clinical tissue samples

In this study, 507 NPC specimens were collected at the Sun Yat-Sen University Cancer Center, Guangzhou, China, between January 2001 and December 2003. The cases were selected based on the following criteria: pathologically confirmed diagnosis of nasopharyngeal carcinoma with biopsy specimens available for tissue microarray (TMA) construction, no previous malignant disease or second primary tumor and no history of radiotherapy, chemotherapy or surgical treatment. All selected samples comprised at least 70% carcinoma tissue as determined by frozen section examination. The patient characteristics are shown in Table 1. The clinical stage was defined according to the 1992 NPC staging system of China [24]. All patients were treated with standard curative radiotherapy with or without chemotherapy. The follow-up period was defined as the time from diagnosis to the date of death or to the time of censure if the patient was still alive. Disease progression was defined as progressive disease after primary treatment or recurrence (local progression) and/or the development of new distant metastases (distant progression). The Institute Research Medical Ethics Committee of Sun Yat-Sen University granted approval for this study.

**Table 1:** Characteristics of nasopharyngeal carcinoma patients (n = 507)

**Characteristic**	**Nasopharyngeal carcinoma patients: n (%)**
**Sex**	
Female	126 (24.9)
Male	381 (75.1)
**Age (years)**	
Median (range)	46 (19–78)
Mean ± SD	46.38 ± 11.065
**Clinical stage**	
I-II	140 (27.7)
III-IV	367 (72.3)
**Progression**	
No	310 (61.1)
Yes	197 (38.9)
**Death**	
No	341 (67.3)
Yes	166 (32.6)
**Follow-up time (months)**	
Median (range)	68 (3–114)
Mean ± SD	60.81 (25.4)
**WHO histological classification**	
NKUC	364 (71.8)
NKDC	129 (25.4)
KSCC	13 (2.6)
Missing	1 (0.2)

SD, standard deviation; WHO, World Health Organization; NKUC, non-keratinizing undifferentiated carcinoma; NKDC, non-keratinizing differentiated carcinoma; KSCC, keratinizing squamous cell carcinoma.

### Tissue microarray construction

The paraffin-embedded specimens were included in the previously constructed tissue microarray (TMA) and the procedures for the TMA construction have been described previously [25]. Briefly, the paraffin-embedded tissue blocks and the corresponding histological H&E-stained slides were overlaid for tissue TMA sampling. Duplicate 1.0 mm diameter cylinders were punched from representative tumor areas of an individual donor tissue block and re-embedded into a recipient paraffin block at a defined position using a tissue array instrument (Beecher Instruments, Silver Spring, MD).

### Immunohistochemistry (IHC) and evaluation

IHC was performed to examine the Hsp70 and HLA-A expression in nasopharyngeal carcinoma tissues, by using primary antibodies against Hsp70 (1:200 dilution; sc-24, Santa Cruz, USA) and HLA-A (1:300 dilution; sc-23446, Santa Cruz, USA), and according to the previous procedures [25]. The IHC results were evaluated and scored independently by three pathologists without knowledge of the patient’s clinicopathological outcomes. A semi-quantitative estimate was made using a composite score obtained by adding the intensity of the staining and the relative abundance of positive cells. The intensity was graded as 0 (negative), 1 (weakly positive), 2 (moderately positive) or 3 (strongly positive). The abundance of positive cells was graded from 0 to 4 (0, < 5% positive cells; 1, 5–25%; 2, 26–50%; 3, 51–75%; 4, 76–100%). The pathologists were in agreement for approximately 82.5% of the cases, which demonstrated that this scoring method was highly reproducible. If at least 2 scores were in agreement, the consensus value was selected. If all three pathologists proposed different results, the pathologists reached a consensus.

### Selection of cutoff score

Receiver operating characteristic (ROC) curve analysis was performed to determine the cutoff score for a “high expression” designation with the 0,1-criterion implemented in SPSS software [25]. First, the clinicopathological characteristics were dichotomized as following groups: T classification (T1-T2 versus T3-T4), N classification (N0 versus N1-N3), clinical stage (I-II versus III-IV), cancer progression (Yes versus No) and survival status (death due to NPC versus censored). Second, the expression scores for Hsp70 were trained in the ROC analysis. The cutoff score is the point on the curve that has both maximum sensitivity and specificity [25].

### Statistical analyses

Statistical analyses were performed with the SPSS statistical software package (standard version 16.0; SPSS, Chicago, IL). Associations between the Hsp70 expression and clinicopathological parameters were assessed using a Chi-Square test. Associations between the Hsp70 expression patterns and HLA-A levels were examined with Pearson correlation and independent *t*-test. Survival curves were plotted by Kaplan-Meier analysis and compared by the log-rank test. The Cox proportional hazards regression model was employed to identify independent prognostic factors. Difference was considered as significant if the *P*-value from a two-tailed test was less than 0.05.

## Results

### Patient characteristics

Among the 507 patients, 381 (75.1%) were male, and the median age was 46 years (ranging 17–78 years). One hundred and twenty-nine tumors (25.4%) were diagnosed as non-keratinizing differentiated carcinoma (NKDC), and 364 tumors (71.8%) were diagnosed as non-keratinizing undifferentiated carcinoma (NKUC), 13 tumors (2.6%) were categorized as keratinizing squamous cell carcinoma (KSCC). One hundred and forty patients (27.7%) were at stages I or II, and 367 patients (72.3%) were at stages III or IV. The median follow-up time was 60.81 months (ranging 3–114 months). Of the total number of patients, 166 (32.6%) died, and 197 (38.9%) experienced disease progression during the five-year follow-up. The detailed clinical information is shown in Table 1.

### Different expression patterns of Hsp70 in NPC

**Figure 1:**
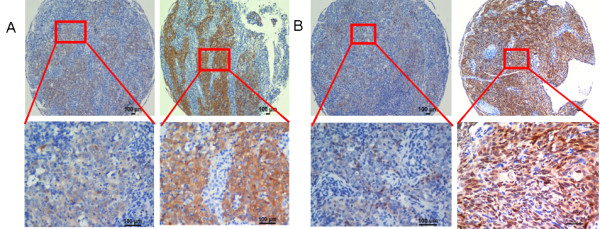
**Different expression patterns of Hsp70 in NPC tumor tissues.A**, Low and high expression levels of Hsp70 in the membrane and cytoplasm in the TMA are shown under both low and high magnification. **B**, Low and high levels of Hsp70 nuclear abundance in the TMA are shown under both low and high magnification. Scale bars, 100 μm.

A total of 507 NPC cases with five-year follow-up information were examined for the Hsp70 expression. Specific Hsp70 staining was detected in the tumor nest cells but not in the stroma. Interestingly, different staining patterns of Hsp70 were observed in NPC tumor tissues (Figure 1 A, B), with spatial difference among nuclear, membrane and cytoplasm. Therefore, the membranal and the cytoplasmic expression or nuclear abundance of Hsp70 were separately scored and evaluated for their predictive values in NPC.

### Selection of the cutoff score for high expression of Hsp70

**Figure 2:**
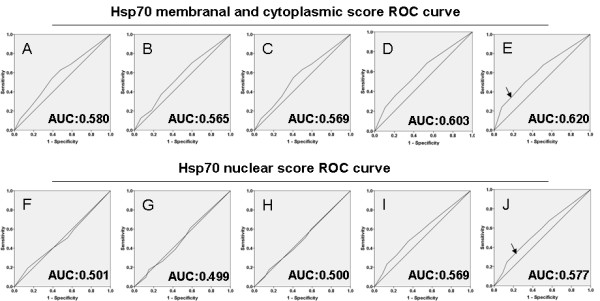
**Selection of the cutoff scores.** Receiver operating characteristic curve analysis was employed to determine the cutoff score for the designation of “high expression” of Hsp70 with membranal, cytoplasmic or nuclear staining. The sensitivity and specificity for each outcome of Hsp70 membranal and cytoplasmic staining were plotted: ( **A**) T classification ( *P* =0.002); ( **B**) N classification ( *P* =0.029); ( **C**) Clinical stage ( *P* =0.029); ( **D**) Cancer progression ( *P* <0.001); ( **E**) Survival status ( *P* <0.001). The sensitivity and specificity for each outcome of Hsp70 nuclear staining were plotted: ( **F**) T classification ( *P* =0.964); ( **G**) N classification ( *P* =0.97); ( **H**) Clinical stage ( *P* =0.99); (I) Cancer progression ( *P* =0.009); (J) Survival status ( *P* =0.005).

ROC curve analysis showed that both extra-nucleic and nucleic abundance of Hsp70 have some sort of predictive values in NPC, with maximum area under curve (AUC) reaching 0.620 and 0.577 for Hsp70 abundance in the two locations, respectively (Figure 2E and J, black arrowed). The points in curves with maximum specificity and sensitivity were treated as cutoff points for high expression of Hsp70. For survival analysis the cutoff score for membranal and cytoplasmic expression of Hsp70 was 3.5, and the cutoff score for the nuclear expression of Hsp70 was defined as 4.5 (Table 2). As a result, tumors designated as low expression of Hsp70 are those with the scores below or equal to the cutoff score, while tumors of high expression are those with scores above the cutoff score.

**Table 2:** The corresponding cutoff score of Hsp70 expression patterns for each clinicopathological feature according to ROC curve analysis

**Feature**	**Hsp70 membranal and cytoplasmic staining**		**Hsp70 nuclear staining**	
	**Cutoff score**	***P* value**	**Cutoff score**	***P* value**
T classification	3.5	0.002	5.5	0.964
N classification	1	0.029	3.5	0.97
Clinical stage	3.5	0.029	5.5	0.99
Cancer progression	1	<0.001	3.5	0.009
Survival status	3.5	<0.001	4.5	0.005

Hsp70, heat shock protein 70; ROC, receiver operating characteristic.

### Association of Hsp70 expression patterns with clinicopathologic features

High membranal and cytoplasmic expression levels of Hsp70 were detected in 226/507 (44.6%) NPC tissues, and high nuclear abundance of Hsp70 were observed in 137/507 (27.1%) NPC tissues. The frequencies of different Hsp70 expression patterns with respect to several clinicopathologic features are detailed in Table 3. Interestingly, we found that high membranal and cytoplasmic expression levels of Hsp70 were significantly negatively correlated with tumor T classification, recurrence or metastasis and advanced clinical stage (*P* < 0.01, Table 3), but no significant association was observed between Hsp70 membranal and cytoplasmic expression levels and other clinicopathologic features such as sex, age and N classification (*P* > 0.05, Table 3). In contrast, high abundance of nucleic Hsp70 was positively correlated with tumor recurrence or metastasis (*P* < 0.05, Table 3), but not with other clinicopathologic features such as tumor T and N classification, sex, age and clinical stage (*P* > 0.05, Table 3).

**Table 3:** Association of different Hsp70 expression patterns and clinicopathological characteristics in NPC patients

**Characteristics**	**Hsp70 membranal and cytoplasmic staining**		***P* value**	**Hsp70 nuclear staining**		***P* value**
	**low expression (n = 281)**	**high expression (n = 226)**		**low expression (n = 370)**	**high expression (n = 137)**	
**Age, years**			0.751			0.857
<46	131	116		176	71	
> = 46	145	115		188	72	
**Sex**			0.828			0.715
Male	209	170		275	104	
Female	72	56		94	34	
**Tumor stage**			0.001			0.401
T1 + T2	96	109		153	51	
T3 + T4	186	116		217	86	
**Lymphoid Nodal(N) state**			0.061			0.468
N0	64	69		94	40	
N1-3	217	157		276	97	
**Recurrence or metastasis**			0.002			0.016
Yes	126	71		132	65	
No	155	155		238	72	
**TNM clinical stage**			0.005			0.900
I + II	63	76		102	37	
III + IV	218	150		268	100	

Hsp70, heat shock protein 70.

### Association between the Hsp70 expression pattern and survival

**Figure 3:**
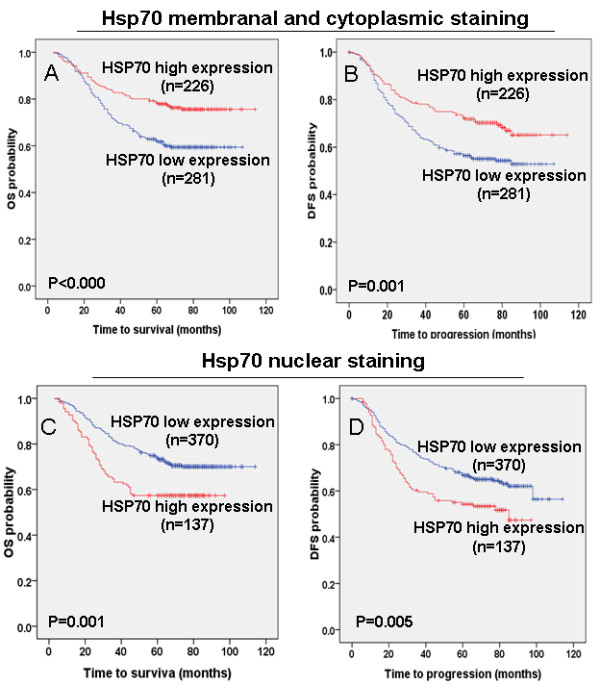
**The association of different Hsp70 expression patterns with NPC patient survival.** TMA analyses of a cohort of 507 NPC patients diagnosed at M0 were conducted. **A** and **B**, high Hsp70 membranal and cytoplasmic expression levels were significantly positively associated with overall survival (OS, *P* = 0.001) and disease-free survival (DFS, *P* = 0.001) in all NPC patients. **C** and **D**, high Hsp70 nuclear abundance were significantly negatively associated with overall survival (OS, *P* = 0.001) and disease-free survival (DFS, *P* = 0.005) in NPC patients.

Due to the finding that the expression of Hsp70 was correlated with recurrence or metastasis, its correlation with survival was examined. The 5-year overall survival rate of the 507 NPC patients was 66.18%. The low and high Hsp70 expression groups were divided by the cutoff value as previously reported [25,26]. For these 507 NPC patients, Kaplan-Meier and log-rank test analyses indicated that patients with high Hsp70 membranal and cytoplasmic expression levels had a significantly better overall survival (OS; 5-year survival rates, 76.1% vs. 60.1%, *P* = 0.001, Figure 3A) and disease-free survival (DFS; 5-year survival rates, 68.6% vs. 55.2%, *P* = 0.001, Figure 3B) than patients with low Hsp70 expression levels. Further stratification analysis by clinical stage revealed that the Hsp70 membranal and cytoplasmic expression levels were not related to OS and DFS in the early stages (Additional file 1: Figure 1A and B); however, for the patients at late-stage, high membranal and cytoplasmic expression levels of Hsp70 were correlated with improved OS (5-year survival rates, 66.7% vs. 52.3%, *P* = 0.006, Additional file 1: Figure 1C) and DFS (5-year survival rates, 61.3% vs. 47.7%, *P* = 0.007, Additional file 1: Figure S1D).

Moreover, the correlation between Hsp70 abundance in the nuclei and survival was tested. Surprisingly, we found that high nuclear abundance of Hsp70 correlated with worse OS (5-year survival rates, 57.7% vs. 70.8%, *P* = 0.001, Figure 3C) and DFS (5-year survival rates, 52.6% vs. 64.3%, *P* = 0.005, Figure 3D) than did low levels of nuclear abundance of Hsp70. Subsequent stratification analysis according to clinical stages revealed that the nucleic abundance of Hsp70 is consistently correlated with worse OS (5-year survival rates, 47% vs. 62.3%, *P* = 0.001, Additional file 2: Figure 2C) and DFS (5-year survival rates, 45.0% vs. 56.3%, *P* = 0.010, Additional file 2:Figure 2D); however, no correlation was found for sample in the early stages (Additional file 2: Figure 2A and B).

### Association between clinicopathologic features, Hsp70 expression patterns and NPC patient survival: univariate and multivariate survival analyses

To confirm the representative of the present NPC cohort, we tested the associations of some well-known prognostic indicators with survival. The univariate Cox proportional hazard regression analysis showed that the clinicopathologic parameters including T classification, N classification, recurrence or metastasis, and clinical stage are each significantly associated with survival (*P* < 0.001; Table 4), which is consistent with the previous findings. Moreover, the assessment of NPC patient survival revealed that high membranal and cytoplasmic expression levels of Hsp70 were significantly correlated with improved overall survival (*P* < 0.001; Table 4), whereas high nuclear abundance of Hsp70 were significantly correlated with poor overall survival (*P* = 0.001, Table 4). In addition, the age was also associated with survival (*P* = 0.002).

**Table 4:** Univariate and multivariate Cox regression analyses of different prognostic variables for NPC patients

**Variable**	**Subset**	**Hazard ratio (95%) CI**	***P*value**
Univariate analysis (n = 507)			
Hsp70 membranal and cytoplasmic staining	low vs. high	0.540 (0.390-0.747)	<0.001
Hsp70 nucleus staining	low vs. high	1.708 (1.241-2.351)	0.001
Age	<46 vs. > = 46	1.644 (1.202-2.249)	0.002
Sex	male vs. female	0.827 (0.574-1.192)	0.308
T stage	T1 + T2 vs. T3 + T4	3.149 (2.159-4.594)	<0.001
N stage	N0 vs. N1 + N2 + N3	2.474 (1.592-3.846)	<0.001
Recurrence or metastasis	No vs. Yes	548.914 (76.728-3.927E3)	<0.001
Clinical stage	I + II vs. III + IV	6.034 (3.352-10.862)	<0.001
Multivariate analysis (n = 507)			
Hsp70 cytoplasmic and membranal staining	low vs. high	0.529 (0.360-0.778)	0.001
Hsp70 nuclear staining	low vs. high	2.601 (1.785-3.789)	6.41E-07
Clinical stage	I + II vs. III + IV	3.641 (1.623-8.170)	0.002

CI, confidence interval; WHO, World Health Organization; Hsp70, heat shock protein 70.

Independency tests were carried out among the prognostic factors, including the different expression patterns of Hsp70 and other significant clinicopathologic features (age, T classification, N classification, distant metastasis and clinical stage), under a multivariate Cox proportional hazards regression analysis (Table 4). We found that high membranal and cytoplasmic expression levels of Hsp70 were independent favorable factors for overall patient survival (hazard ratio: 0.529; 95% confidence interval: 0.360–0.778; *P* = 0.001), whereas high nuclear levels of Hsp70 were an independent risk factor for overall patient survival (hazards ratio: 2.601; 95% confidence interval: 1.785–3.789; *P* < 0.001). Among the other variables, clinical stage was also found to be an independent prognostic predictor of overall survival (Table 4).

### Correlation between the Hsp70 abundance and the expression of HLA-A

**Figure 4:**
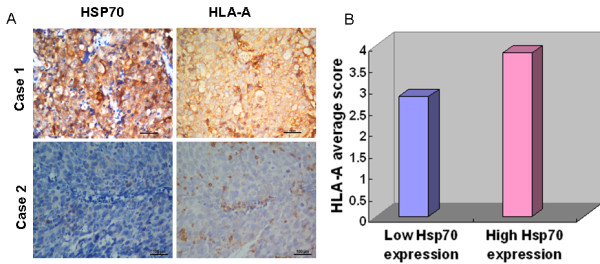
**Correlation between expression levels of Hsp70 and the level of HLA-A in NPC tissues.A**, continuous sections of human NPC tissue were subjected to IHC staining with antibodies against Hsp70 and HLA-A. The high membranal and cytoplasmic expression levels of Hsp70 in the tumor tissue in case 1 were accompanied by an elevated level of HLA-A. Conversely, the low membranal and cytoplasmic expression levels of Hsp70 in the tumor tissue of case 2 were accompanied by a low level of HLA-A. Scale bars = 100 μm. **B**, in 226 NPC cases with high membranal and cytoplasmic expression levels of Hsp70, an average HLA-A score was 3.82 ( *right column*), an average score that was significantly higher than that (2.81) of the 281 NPCs with low expression levels of Hsp70 ( *left column*, *P* < 0.001, independent sample *t-*test).

In light of the involvement of Hsp70 in the antigen presentation process, which HLA-A plays important role, we further investigated the correlation between its abundance and HLA-A expression. The results showed a markedly positive correlation between the membranal and cytoplasmic expression level of Hsp70 and the expression of HLA-A in NPC tissues (Figure 4A) (Pearson Correlation = 0.255). In the 226 NPC samples with high expression of membranal and cytoplasmic Hsp70, the average HLA-A score was 3.82, which was significantly higher than that (2.81) found in the remaining 281 NPCs with low expression of membranal and cytoplasmic Hsp70 (*P* < 0.001, independent sample *t*-test; Figure 4B). On the other hand, although low expression of HLA-A was found in NPC tumors with abundant nucleic Hsp70 (Pearson Correlation = −0.145), the correlation didn’t reach a statistically significance (*p* = 0.092).

## Discussion

As a member of the HSP family, Hsp70 has been considered as cancer relevant protein. On one hand, its high expression has been demonstrated in malignant tumors such as breast cancer [27], lung cancer [28], oral squamous cell carcinoma [29] and prostate cancer [30]; and moreover, its overexpression has been correlated with poor prognosis and resistance to therapy [27-30]. These suggest that Hsp70 might promote somehow tumor development. On the other hand, previous studies have shown that Hsp70 acts as chaperone for presenting tumor antigens to activate immune response [20,21], and mediate anti-cancer immunity [31], suggesting the opposite role of Hsp70 to tumor development. The mechanism underlying the dual roles of Hsp70 to tumorigenesis is still unclear.

In the present study, the expression of Hsp70 was determined by IHC in a large cohort of NPC tissues using a NPC-TMA with follow-up data. Among the 507 patients, we noted distinct nuclear, membranal and cytoplasmic localization of Hsp70 in NPC tissues. Interestingly, we observed that distinct Hsp70 expression patterns correlated with different clinical outcomes. High membranal and cytoplasmic expression levels of Hsp70 predicted an improved survival rate, whereas high nuclear abundance of Hsp70 was correlated with a poor survival rate. These findings suggest that Hsp70 might be a favorable prognostic indicator, which could be used in the search of novel treatment to improve survival for the patient with poor predicted outcome.

More importantly, further correlation analyses revealed that high membranal and cytoplasmic expression levels of Hsp70 in NPCs were negatively associated with tumor T classification, N classification, recurrence and metastasis, advanced clinical stage and histological type. In contrast, high nuclear abundance of Hsp70 was positively correlated with tumor recurrence, metastasis and histological type. These results suggested that membranal and cytoplasmic Hsp70 might play an anti-tumor role, whereas nuclear Hsp70 may provide a selective advantage in the NPC tumorigenic processes.

NPC is a malignancy widely known as Epstein-Barr virus (EBV)-related, and thus many immune-related genes have been examined for the association with NPC development. Among them, human leukocyte antigen (HLA) has been extensively studied, considering the capability of HLA to present antigen to cytotoxic T cells and, thus, trigger the host immune response against viral infection. Genetic studies have shown that some HLA alleles such as HLA-A*0203 and HLA-A*0207 are risk alleles while HLA-A*1101 is a protective allele for NPC [32]. Moreover, the genome-wide expression profiling study showed that the expression of MHC class I molecules were inhibited by EBV genes in NPC tumors [33]. HLA restricted EBV variants, which lose T-cell epitopes have been demonstrated in NPC [34,35]. In addition, loss of HLA locus in NPC tumor genome was observed (unpublished data). These findings consistently support the contribution of HLA to NPC development. In this study, HLA-A expression was found in NPC tumors, and was positively correlated with the membranal and cytoplasmic expression levels of Hsp70, which are in agreement with previous studies [36,37]. Hsp70 family members are known as facilitators of immune responses by interacting with receptor on antigen-presenting cells leading to Hsp70-peptide uptake and antigen cross priming [38]. Moreover, As a chaperone, Hsp70 has the abilities to induce strong antiviral T-cell responses [39] and to elicit LMP2A-specific IFN-γ-producing cells and rousted cytotoxic T lymphocytes (CTLs) *in vitro* and *in vivo*[40]. Furthermore, it has been shown that HSP70 significantly increase MHC class I cell surface expression and antigen presentation on melanoma B16 cells [41,42]. Therefore, the membranal and cytoplasmic Hsp70 might play a role in the HLA related immune response, so as to exhibit the anti-tumor effect on NPC. Indeed, the application of Hsp70 for cancer therapy as a tumor-antigen carrier has been extensively investigated in cancers [22,43-47], including NPC [23].

On the other hand, we found high Hsp70 abundance in the nuclei in NPC tissues, which positively correlated with tumor recurrence, distant metastasis and a poor survival rate. Moreover, in NPC tumors with Hsp70 abundant in the nuclear, we found low abundance of HLA-A, even though the correlation didn’t reach a statistically significance. These findings suggested that nucleic localization of Hsp70 might decrease HLA-A cell surface expression and antigen presentation, and play tumorigenic role in NPC. This is supported by previous report that Hsp70 can effectively inhibit tumor cell death induced by several stresses such as heat shock, hypoxia and oxidative stress [48]. Moreover, studies have shown that Hsp70 can translocate from cytoplasm to nucleus or nucleoli under heat-stress conditions and plays a protective role against stress-mediated apoptosis and DNA damage by co-localizing with DNA repair proteins, [49,50], and thus increases tumor cell survival.

## Conclusion

Here, we described the distinct expression patterns of Hsp70 in human NPC tissues, which might play different roles during NPC tumor cell progression, could predict different patient outcomes for the first time. Furthermore, our data suggest that the different localization of Hsp70 could be molecular targets for the development of novel therapeutic strategies.

## Abbreviations

Hsp70: heat shock protein 70; IHC: immunohistochemistry; NPC: nasopharyngeal carcinoma; APCs: antigen-presenting cells; TMA: tissue microarray; ROC: receiver operating characteristic; NKDC: non-keratinizing differentiated carcinoma; NKUC: non-keratinizing undifferentiated carcinoma; KSCC: keratinizing squamous cell carcinoma; AUC: areas under the curve.

## Competing interests

The authors declare that they have no competing interests.

## Authors’ contributions

MB conducted the study, participated in the data collection, performed most experiments, and wrote the initial draft and revised the manuscripts. XP and JX collected the preliminary data, and helped to perform some experiments. HQ, CC and TB participated in the study design and interpretation of the data. RJ, YL, QS, HY, LZ and SF undertaking immunohistochemistry work. JX, JY and YX study coordination and revision of the paper. All authors read and approved the final manuscript.

## Supplementary Material

Additional file 1 **Figure S1.** Association of Hsp70 membranal and cytoplasmic expression levels with survival of NPC patients at different stages. A and B, no significant differences in five-year OS and DFS rates were found between low and high levels of Hsp70 membranal and cytoplasmic expression levels in NPC patients with early stage disease (stage I - II). C and D, high Hsp70 membranal and cytoplasmic expression levels were significantly positively associated with overall survival (OS, *P* = 0.006) and disease-free survival (DFS, *P* = 0.007) in NPC patients with late-stage disease (stage III-IV). Click here for file

Additional file 2 **Figure S2.** Association of Hsp70 nuclear abundance with survival of NPC patients at different stages. A and B, no significant differences in five-year OS and DFS rates were found between low and high levels of Hsp70 nuclear abundance in NPC patients with early stage disease (stage I - II). C and D, high Hsp70 nuclear abundance were significantly positively associated with the survival (OS, *P* = 0.006) and disease-free survival (DFS, *P* = 0.007) in NPC patients with late-stage disease (stage III-IV). Click here for file

## References

[B1] Wei WI Sham JS Nasopharyngeal carcinoma Lancet 2005 365 9476 2041 2054 10.1016/S0140-6736(05)66698-6 15950718

[B2] Chang ET Adami HO The enigmatic epidemiology of nasopharyngeal carcinoma Cancer Epidemiol Biomarkers Prev 2006 15 10 1765 1777 10.1158/1055-9965.EPI-06-0353 17035381

[B3] Klibi J Niki T Riedel A Pioche-Durieu C Souquere S Rubinstein E Le Moulec S Guigay J Hirashima M Guemira F Adhikary D Mautner J Busson P Blood diffusion and Th1-suppressive effects of galectin-9-containing exosomes released by Epstein-Barr virus-infected nasopharyngeal carcinoma cells Blood 2009 113 9 1957 1966 10.1182/blood-2008-02-142596 19005181

[B4] Baujat B Audry H Bourhis J Chan AT Onat H Chua DT Kwong DL Al-Sarraf M Chi KH Hareyama M Pignon JP MAC-NPC Collaborative Group Chemotherapy in locally advanced nasopharyngeal carcinoma: an individual patient data meta-analysis of eight randomized trials and 1753 patients Int J Radiat Oncol Biol Phys 2006 64 1 47 56 10.1016/j.ijrobp.2005.06.037 16377415

[B5] Al-Sarraf M LeBlanc M Giri PG Fu KK Cooper J Vuong T Forastiere AA Adams G Sakr WA Schuller DE Ensley JF Chemoradiotherapy versus radiotherapy in patients with advanced nasopharyngeal cancer: phase III randomized Intergroup study 0099 J Clin Oncol 1998 16 4 1310 1317 9552031 10.1200/JCO.1998.16.4.1310

[B6] Langendijk JA Leemans CR Buter J Berkhof J Slotman BJ The additional value of chemotherapy to radiotherapy in locally advanced nasopharyngeal carcinoma: a meta-analysis of the published literature J Clin Oncol 2004 22 22 4604 4612 10.1200/JCO.2004.10.074 15542811

[B7] Chan SL Ma BB Novel systemic therapeutic for nasopharyngeal carcinoma Expert Opin Ther Targets 2012 16 Suppl 1 S63 S68 22313344 10.1517/14728222.2011.635646

[B8] Komatsu M Tsukuda M Matsuda H Horiuchi C Taguch T Takahashi M Nishimura G Mori M Niho T Ishitoya J Sakuma Y Hirama M Shiono O Comparison of concurrent chemoradiotherapy versus induction chemotherapy followed by radiation in patients with nasopharyngeal carcinoma Anticancer Res 2012 32 2 681 686 22287763

[B9] Patel SG Shah JP TNM staging of cancers of the head and neck: striving for uniformity among diversity CA Cancer J Clin 2005 55 6 242 258 16020425 10.3322/canjclin.55.4.242

[B10] Ma BB Chan AT Recent perspectives in the role of chemotherapy in the management of advanced nasopharyngeal carcinoma Cancer 2005 103 1 22 31 10.1002/cncr.20768 15565580

[B11] Chua DT Ma J Sham JS Mai HQ Choy DT Hong MH Lu TX Min HQ Long-term survival after cisplatin-based induction chemotherapy and radiotherapy for nasopharyngeal carcinoma: a pooled data analysis of two phase III trials J Clin Oncol 2005 23 6 1118 1124 10.1200/JCO.2005.12.081 15657403

[B12] Hockel M Vaupel P Biological consequences of tumor hypoxia Semin Oncol 2001 28 2 Suppl 8 36 41 11395851

[B13] Cui X Yu ZY Wang W Zheng YQ Liu W Li LX Co-Inhibition of HSP70/HSP90 Synergistically Sensitizes Nasopharyngeal Carcinoma Cells to Thermotherapy Integr Cancer Ther 2011 [Epub ahead of print] 10.1177/1534735411399900 21498475

[B14] Gehrmann M Radons J Molls M Multhoff G The therapeutic implications of clinically applied modifiers of heat shock protein 70 (Hsp70) expression by tumor cells Cell Stress Chaperones 2008 13 1 1 10 10.1007/s12192-007-0006-0 18347936 PMC2666213

[B15] Wei YQ Zhao X Kariya Y Teshigawara K Uchida A Inhibition of proliferation and induction of apoptosis by abrogation of heat-shock protein (HSP) 70 expression in tumor cells Cancer Immunol Immunother 1995 40 2 73 78 10.1007/BF01520287 7882385 PMC11037574

[B16] Jaattela M Wissing D Kokholm K Kallunki T Egeblad M Hsp70 exerts its anti-apoptotic function downstream of caspase-3-like proteases EMBO J 1998 17 21 6124 6134 10.1093/emboj/17.21.6124 9799222 PMC1170939

[B17] Rerole AL Jego G Garrido C Hsp70: anti-apoptotic and tumorigenic protein Methods Mol Biol 2011 787 205 230 10.1007/978-1-61779-295-3_16 21898238

[B18] Tong YQ Zhang ZJ Liu B Huang J Liu H Liu Y Guo FJ Zhou GH Xie PL Li YH Zuo CH Hu JY Li GC Autoantibodies as potential biomarkers for nasopharyngeal carcinoma Proteomics 2008 8 15 3185 3193 10.1002/pmic.200700651 18654982

[B19] Liao Q Zhao L Chen X Deng Y Ding Y Serum proteome analysis for profiling protein markers associated with carcinogenesis and lymph node metastasis in nasopharyngeal carcinoma Clin Exp Metastasis 2008 25 4 465 476 10.1007/s10585-008-9152-8 18357507 PMC2413104

[B20] Calderwood SK Theriault JR Gong J How is the immune response affected by hyperthermia and heat shock proteins? Int J Hyperthermia 2005 21 8 713 716 10.1080/02656730500340794 16338853

[B21] Srivastava PK Immunotherapy for human cancer using heat shock protein-peptide complexes Curr Oncol Rep 2005 7 2 104 108 10.1007/s11912-005-0035-8 15717943

[B22] Srivastava PK Menoret A Basu S Binder RJ McQuade KL Heat shock proteins come of age: primitive functions acquire new roles in an adaptive world Immunity 1998 8 6 657 665 10.1016/S1074-7613(00)80570-1 9655479

[B23] Liu G Yao K Wang B Zhou F Chen Y Li L Chi J Peng G Reconstituted complexes of mycobacterial HSP70 and EBV LMP2A-derived peptides elicit peptide-specific cytotoxic T lymphocyte responses and anti-tumor immunity Vaccine 2011 29 45 7414 7423 21807054 10.1016/j.vaccine.2011.07.063

[B24] Min H Hong M Ma J Zhang E Zheng Q Zhang J Zhang F Su Y Qiu F A new staging system for nasopharyngeal carcinoma in China Int J Radiat Oncol Biol Phys 1994 30 5 1037 1042 10.1016/0360-3016(94)90307-7 7961009

[B25] Wang HY Sun BY Zhu ZH Chang ET To KF Hwang JS Jiang H Kam MK Chen G Cheah SL Lee M Liu ZW Chen J Zhang JX Zhang HZ He JH Chen FL Zhu XD Huang MY Liao DZ Fu J Shao Q Cai MB Du ZM Yan LX Hu CF Ng HK Wee JT Qian CN Liu Q Ernberg I Ye W Adami HO Chan AT Zeng YX Shao JY Eight-Signature Classifier for Prediction of Nasopharnyngeal Carcinoma Survival J Clin Oncol 2011 29 34 4516 4525 10.1200/JCO.2010.33.7741 22025164

[B26] Cai MY Tong ZT Zhu W Wen ZZ Rao HL Kong LL Guan XY Kung HF Zeng YX Xie D H3K27me3 Protein Is a Promising Predictive Biomarker of Patients’ Survival and Chemoradioresistance in Human Nasopharyngeal Carcinoma Mol Med 2011 [Epub ahead of print] 10.2119/molmed.2011.00054 PMC3321814 21738951

[B27] Boroughs LK Antonyak MA Johnson JL Cerione RA A unique role for heat shock protein 70 and its binding partner tissue transglutaminase in cancer cell migration J Biol Chem 2011 286 43 37094 37107 10.1074/jbc.M111.242438 21896482 PMC3199457

[B28] Malusecka E Krzyzowska-Gruca S Gawrychowski J Fiszer-Kierzkowska A Kolosza Z Krawczyk Z Stress proteins HSP27 and HSP70i predict survival in non-small cell lung carcinoma Anticancer Res 2008 28 1B 501 506 18383892

[B29] Thubashini M Malathi N Kannan L Expression of heat shock protein70 in oral submucous fibrosis and oral squamous cell carcinoma: an immunohistochemical study Indian J Dent Res 2011 22 2 256 259 10.4103/0970-9290.84299 21891896

[B30] Lu S Tan Z Wortman M Dong Z Regulation of heat shock protein 70–1 expression by androgen receptor and its signaling in human prostate cancer cells Int J Oncol 2010 36 2 459 467 20043082 PMC2929386

[B31] Zeng Y Chen X Larmonier N Larmonier C Li G Sepassi M Marron M Andreansky S Katsanis E Natural killer cells play a key role in the antitumor immunity generated by chaperone-rich cell lysate vaccination Int J Cancer 2006 119 11 2624 2631 10.1002/ijc.22150 16989012

[B32] Bei JX Jia WH Zeng YX Familial and large-scale case–control studies identify genes associated with nasopharyngeal carcinoma Semin Cancer Biol 2012 22 2 96 106 10.1016/j.semcancer.2012.01.012 22313875

[B33] Sengupta S den Boon JA Chen IH Newton MA Dahl DB Chen M Cheng YJ Westra WH Chen CJ Hildesheim A Sugden B Ahlquist P Genome-wide expression profiling reveals EBV-associated inhibition of MHC class I expression in nasopharyngeal carcinoma Cancer Res 2006 66 16 7999 8006 10.1158/0008-5472.CAN-05-4399 16912175

[B34] Tang YL Lu JH Cao L Wu MH Peng SP Zhou HD Huang C Yang YX Zhou YH Chen Q Li XL Zhou M Li GY Genetic variations of EBV-LMP1 from nasopharyngeal carcinoma biopsies: potential loss of T cell epitopes Braz J Med Biol Res 2008 41 2 110 116 18297191 10.1590/s0100-879x2008000200006

[B35] Lin JC Cherng JM Lin HJ Tsang CW Liu YX Lee SP Amino acid changes in functional domains of latent membrane protein 1 of Epstein-Barr virus in nasopharyngeal carcinoma of southern China and Taiwan: prevalence of an HLA A2-restricted ‘epitope-loss variant’ J Gen Virol 2004 85 7 2023 2034 10.1099/vir.0.19696-0 15218188

[B36] Maranon C Egui A Carrilero B Thomas MC Pinazo MJ Gascon J Segovia M Lopez MC Identification of HLA-A *02:01-restricted CTL epitopes in Trypanosoma cruzi heat shock protein-70 recognized by Chagas disease patients Microbes Infect 2011 13 12–13 1025 1032 21704723 10.1016/j.micinf.2011.05.010

[B37] Okochi M Hayashi H Ito A Kato R Tamura Y Sato N Honda H Identification of HLA-A24-restricted epitopes with high affinities to Hsp70 using peptide arrays J Biosci Bioeng 2008 105 3 198 203 10.1263/jbb.105.198 18397768

[B38] Mizukami S Kajiwara C Tanaka M Kaisho T Udono H Differential MyD88/IRAK4 requirements for cross-priming and tumor rejection induced by heat shock protein 70-model antigen fusion protein Cancer Sci 2012 Epub ahead of print 10.1111/j.1349-7006.2012.02233.x PMC7659281 22320267

[B39] Tischer S Basila M Maecker-Kolhoff B Immenschuh S Oelke M Blasczyk R Eiz-Vesper B Heat shock protein 70/peptide complexes: potent mediators for the generation of antiviral T cells particularly with regard to low precursor frequencies J Transl Med 2011 9 175 10.1186/1479-5876-9-175 21992180 PMC3217864

[B40] Liu G Yao K Wang B Zhou F Chen Y Li L Chi J Peng G Reconstituted complexes of mycobacterial HSP70 and EBV LMP2A-derived peptides elicit peptide-specific cytotoxic T lymphocyte responses and anti-tumor immunity Vaccine 2011 29 43 7414 7423 10.1016/j.vaccine.2011.07.063 21807054

[B41] Wells AD Rai SK Salvato MS Band H Malkovsky M Hsp72-mediated augmentation of MHC class I surface expression and endogenous antigen presentation Int Immunol 1998 10 5 609 617 10.1093/intimm/10.5.609 9645609

[B42] Dressel R Lubbers M Walter L Herr W Gunther E Enhanced susceptibility to cytotoxic T lymphocytes without increase of MHC class I antigen expression after conditional overexpression of heat shock protein 70 in target cells Eur J Immunol 1999 29 12 3925 3935 10.1002/(SICI)1521-4141(199912)29:12<3925::AID-IMMU3925>3.0.CO;2-S 10602000

[B43] Kumar S Deepak P Acharya A Autologous Hsp70 immunization induces anti-tumor immunity and increases longevity and survival of tumor-bearing mice Neoplasma 2009 56 3 259 268 10.4149/neo_2009_03_259 19309230

[B44] Tamura Y Peng P Liu K Daou M Srivastava PK Immunotherapy of tumors with autologous tumor-derived heat shock protein preparations Science 1997 278 5335 117 120 10.1126/science.278.5335.117 9311915

[B45] Srivastava PK Immunotherapy of human cancer: lessons from mice Nat Immunol 2000 1 5 363 366 10.1038/80795 11062489

[B46] Parmiani G Testori A Maio M Castelli C Rivoltini L Pilla L Belli F Mazzaferro V Coppa J Patuzzo R Heat shock proteins and their use as anticancer vaccines Clin Cancer Res 2004 10 24 8142 8146 10.1158/1078-0432.CCR-04-1194 15623587

[B47] Nishikawa M Otsuki T Ota A Guan X Takemoto S Takahashi Y Takakura Y Induction of tumor-specific immune response by gene transfer of Hsp70-cell-penetrating peptide fusion protein to tumors in mice Mol Ther 2010 18 2 421 428 10.1038/mt.2009.203 19724264 PMC2839311

[B48] Rohde M Daugaard M Jensen MH Helin K Nylandsted J Jaattela M Members of the heat-shock protein 70 family promote cancer cell growth by distinct mechanisms Genes Dev 2005 19 5 570 582 10.1101/gad.305405 15741319 PMC551577

[B49] Welch WJ Feramisco JR Nuclear and nucleolar localization of the 72,000-dalton heat shock protein in heat-shocked mammalian cells J Biol Chem 1984 259 7 4501 4513 6368558

[B50] Kotoglou P Kalaitzakis A Vezyraki P Tzavaras T Michalis LK Dantzer F Jung JU Angelidis C Hsp70 translocates to the nuclei and nucleoli, binds to XRCC1 and PARP-1, and protects HeLa cells from single-strand DNA breaks Cell Stress Chaperones 2009 14 4 391 406 10.1007/s12192-008-0093-6 19089598 PMC2728274

