# Supplementary figures and images for: Expression of heat shock protein 70 in nasopharyngeal carcinomas: different expression patterns correlate with distinct clinical prognosis
Source: J Transl Med. 2012 May 16;10:96. doi: 10.1186/1479-5876-10-96 (PMC3478221; doi:10.1186/1479-5876-10-96)

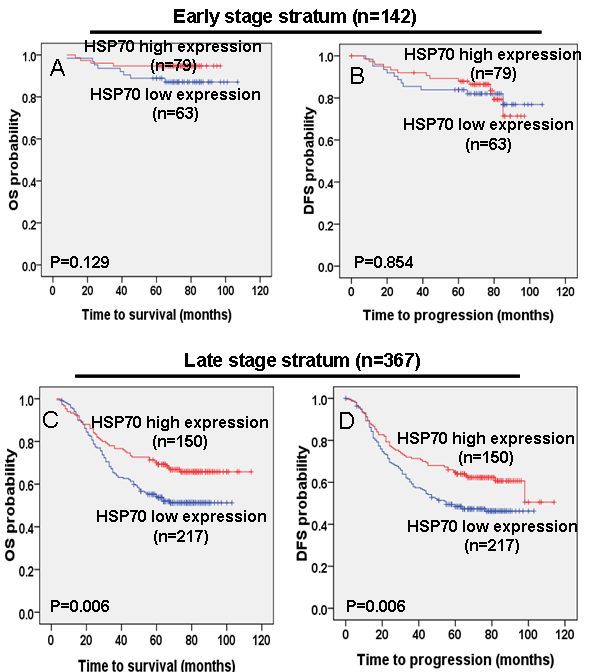

Supplement: Additional file 1 — Figure S1. Association of Hsp70 membranal and cytoplasmic expression levels with survival of NPC patients at different stages. A and B, no significant differences in five-year OS and DFS rates were found between low and high levels of Hsp70 membranal and cytoplasmic expression levels in NPC patients with early stage disease (stage I - II). C and D, high Hsp70 membranal and cytoplasmic expression levels were significantly positively associated with overall survival (OS, P = 0.006) and disease-free survival (DFS, P = 0.007) in NPC patients with late-stage disease (stage III-IV). [file 1479-5876-10-96-S1.tiff]

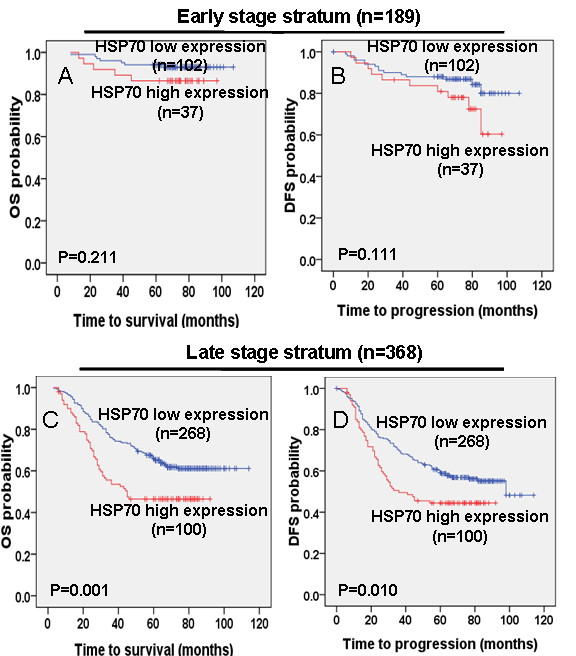

Supplement: Additional file 2 — Figure S2. Association of Hsp70 nuclear abundance with survival of NPC patients at different stages. A and B, no significant differences in five-year OS and DFS rates were found between low and high levels of Hsp70 nuclear abundance in NPC patients with early stage disease (stage I - II). C and D, high Hsp70 nuclear abundance were significantly positively associated with the survival (OS, P = 0.006) and disease-free survival (DFS, P = 0.007) in NPC patients with late-stage disease (stage III-IV). [file 1479-5876-10-96-S2.tiff]
